# Efficacy of Probiotic Tablets in the Reduction of Halitosis: A Randomised, Single Blind, Controlled Clinical Trial

**DOI:** 10.3290/j.ohpd.b5866901

**Published:** 2024-12-03

**Authors:** Guixia Huang, Nan Li

**Affiliations:** a Guixia Huang Senior Engineer, R&D Department, Sirio Pharma Co. Ltd., Shantou, China. Collected the data, drafted the initial manuscript, conducted the study, statistical analysis, study concept and design, critical revisions of the manuscript.; b Nan Li Senior Engineer, R&D Department, Sirio Pharma Co. Ltd., Shanghai, China. Review and editing, supervised the study.

**Keywords:** fresh breath, halitosis, natural extracts, probiotic, volatile sulphur compounds (VSCs)

## Abstract

**Purpose:**

To evaluate the effect of a fresh-breath mild effervescent tablet on halitosis as an alternative to mouthwash.

**Materials and Methods:**

Halitosis is the unpleasant and offensive odour emanating from the oral cavity (bad breath), which is linked to the presence of volatile sulphur compounds (VSCs). A randomised, single-blind, controlled clinical trial was conducted with 102 volunteers who had oral complaints (range 18–60 years). Breath samples were taken and analysed for the level of hydrogen sulphide (H2S), methyl mercaptan (CH3SH) and dimethyl sulphide (CH3SCH3). Sixty-one volunteers who met the halitosis criteria were enrolled and completed the clinical trial. Two groups were formed according to the test employed: the Immediate Effect Test (IET) (n = 31) and the Effective Duration Test (EDT) (n = 30). In the IET, subjects were divided into three subgroups: fresh-breath mild effervescent tablet (MT, n = 11), the fresh-breath lozenge tablet (LT, n = 10), and fresh-breath mouthwash (MW, n = 10). Halitosis was measured immediately after completely consuming the test tablet or gargling. In the EDT, subjects were also divided into three subgroups: fresh-breath mild effervescent tablet (MT, n = 10), probiotic powder (PP, n = 10) and probiotic tablet (PT, n = 10). Halitosis was measured at 0 h, 1 h, and 2 h after consuming the test substances.

**Results:**

In the IET group, a statistically significant reduction in VSCs was obtained in all three subgroups compared with baseline (VSCs of MT group, p < 0.01, VSCs of LT subgroup, p < 0.05, VSCs of MT group, p < 0.01). The fresh-breath mild effervescent tablet subgroup showed a statistically significantly greater reduction in VSCs compared to the fresh-breath lozenge subgroup (p < 0.01). No statistically significant differences in VSC reduction were found between the fresh-breath mild effervescent tablet subgroup and the mouthwash group (p = 0.38). In the EDT group, the VSC level of the fresh-breath mild effervescent tablet group was still statistically significantly lower than the baseline 2 h after consumption (p < 0.01). The two control groups which used commercial fresh-breath probiotic products did not show any statistically significant difference compared to baseline. The VSC level of these two groups gradually returned to the baseline level 2 h after consumption.

**Conclusion:**

Fresh-breath mild effervescent tablets have shown promising potential as an alternative, effective, and potentially safe choice for fresh breath in comparison to mouthwash.

On November 18, 2022, the World Health Organization (WHO) released its latest Global Status Report on oral health: nearly half of the world’s population (45% or 3.5 billion people) suffer from oral diseases, and three-quarters of them live in low- and middle-income countries.^9^ The number of global cases of oral disease has increased by 1 billion over the past 30 years, indicating that many people lack access to oral disease prevention and treatment services.^9^ Oral diseases have led to enormous health, social and economic burdens. Oral health is listed as one of the ten standards of human health by the World Health Organization and is closely related to general health.^25^ Bad breath (halitosis) has a serious impact on people’s social interaction and mental health, and has been listed as a global disease by the WHO.

Halitosis could have a variety of causes, such as poor oral hygiene and bad habits (tobacco use, excessive use of alcohol), caries, periodontal diseases (periodontitis and gingivitis), retention of food between teeth, and tongue debris. Karbalaei et al^14^ described causes of halitosis: Gram-negative anaerobic bacteria digest proteins from food residues, desquamated cells and the action of leucocytes in the oral mucosa, and salivary debris releasing amino acids that accumulate in the oral cavity. Poor oral hygiene and restoration defects lead to accumulation of food debris and dental bacterial plaque on the teeth and tongue; degradation of this retained debris by bacteria causes oral halitosis. VSCs are derived metabolites of the bacterial putrefaction process.^14^ The majority of VSCs are produced following degradation of food and salivary proteins by oral bacteria, and the use of amino acids by VSC-producing bacteria.^14^ Bacteria such as Porphyromonas gingivalis, Treponema denticola, Prevotella intermedia and Fusobacterium nucleatum are commonly involved in periodontitis.^14^ Volatile sulfur compounds (VSCs) are the major odoriferous components of halitosis, with hydrogen sulfide (H2S), methyl mercaptan (CH3SH), and dimethyl sulfide (CH3SCH3) as the main contributors.^7^

Halitosis can be treated by mechanical or chemical methods, or a combination of both, such as reducing the buildup of bacterial biofilm and food debris, which decreases the quantity of VSC-producing microorganisms in the oral cavity. Alsaffar et al^1^ suggested that the effectiveness of CHX-CPC-Zn mouthwash (containing 0.05% chlorhexidine, 0.05% cetylpyridinium chloride, and 0.14% zinc lactate) in reducing VSC levels could be explained by the combined antibacterial and VSC-neutralising actions. Neutralising VSCs with zinc ions was more effective immediately, while reduction of the microorganisms by the antibacterial properties of the mouthwash was more effective in the long-term.^1^ Some natural plant and fungal extracts have also been reported to have the potential to reduce VSCs. The phenol hydroxyl group of polyphenols is easily dehydrogenated in alkaline solution and becomes a phenoxyl radical. When dissolved in pH-10 alkaline solution, theaflavins in tea showed extremely strong activity, and the effectiveness against methyl mercaptan reached 0.232 mg separately per 1 mg 40% theaflavins.^10^ Mercaptan-capturing properties of 33 kinds of mushrooms were measured by Negishi et al,^20^ reporting high capturing ability toward methyl mercaptan (MeSH) by Agaricus bisporus, A. campestris, and Boletus fraternus. These studies indicated that some natural extracts have good effects in terms of VSC reduction. Kim et al^16^ evaluated the effect of a mouthwash containing Lespedeza cuneata extract (LCE) on halitosis as an alternative to chemical mouthwashes. Those authors found this natural extract to be effective in reducing halitosis both subjectively and objectively, which suggested an antibacterial effect on halitosis-causing bacteria(HCBs) in the oral cavity.

Probiotics, another potential natural tool, have been reported to have beneficial effects on oral health, such as caries,^13^ gingival health,^13^ and halitosis.^11^ Several studies suggest that consumption of products containing probiotics could reduce the number of Streptococcus mutans in saliva.^12,13^ Li et al^19^ reviewed the halitosis research, explored the associated aetiology, and suggested improving oral hygiene habits and regulating oral or intestinal flora with antimicrobials or probiotics as interventions.

Halitosis can negatively impact social life and self-esteem, making it essential to seek ways to prevent it. The articles cited above reported that some natural extracts and probiotics had effects on VSC reduction and regulating oral flora, which may reduce halitosis. Therefore, the aim of this study was to assess the effect of a fresh-breath mild effervescent tablet containing natural extracts and probiotics as effective ingredients on the three major odoriferous components of halitosis: H2S, CH3SH, CH3SCH3 and the total VSCs (the sum of H2S, CH3SH and CH3SCH3).

## MATERIALS AND METHODS

### Study Participants and Eligibility Criteria

The sample size was determined referring to Alsaffar et al,^1^ by assuming an effect size (f) of 0.80, a power of 90% (1−β = 0.90) and α = 0.05. The minimal number of subjects was calculated as 10 to establish a statistically significant difference between different study groups.

**Fig 1:**
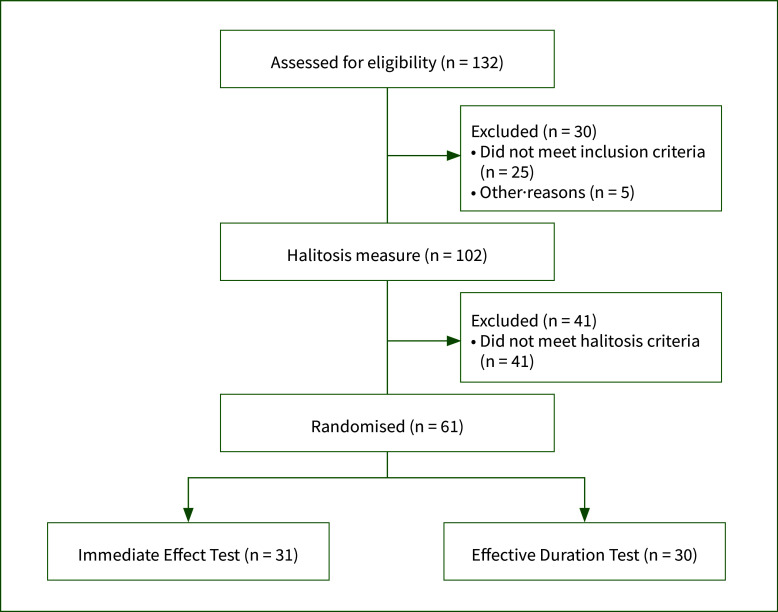
Participants’ enrollment.

132 participants filled out the recruitment questionnaire. Of these, 102 volunteers were chosen to be the potential candidates through the questionnaire according to their oral status and habits, while 25 people were excluded because of smoking, having undergone major oral surgery in the past year, and antibiotic use, and 5 participants were thought to have a low probability of halitosis. A final analysis was conducted on 61 halitosis participants who were randomly selected and divided into the groups Immediate Effect Test ([IET] 31 participants) and Effective Duration Test ([EDT] 30 participants) (Fig 1). Furthermore, oral health and dietary education was provided to ensure homogeneity of oral health behaviour among the participants as much as possible.

#### Inclusion Criteria

Healthy volunteers aged 18–60 years who had dental caries or periodontitis/gingivitis^22^ as diagnosed by a dentist, self-assessed or receiving complaints of bad breath were involved as potential participants. Halitosis was defined as any two VSC levels recorded in parts per billion (ppb) of the breath sample meeting the following thresholds: H2S ≥ 112 ppb (150 ng/l), CH3SH ≥ 26 ppb (50 ng/l), CH3SCH3 ≥ 8 ppb (20 ng/l), total VSCs ≥ 150 ppb.^2,8^ Subjects matching the halitosis criteria were recruited.

#### Exclusion Criteria

Subjects were excluded from this study if they had any of the following conditions: having received major oral surgery in the past year; smokers; antibiotic use in the previous 3 months; unable to use the samples as required or incomplete information; other reasons making them unsuitable for clinical trials.

### Study Protocol

This study was conducted as a randomised, single-blind, controlled clinical trial. An experienced scientist (GH.; Good Clinical Practice certified) carried out the study and explained the study objective directly to the participants, and also gave them instructions about behaviour and diet to avoid affecting the study. Halitosis was measured at the baseline, and participants who met the halitosis criteria were enolled. The enrolled subjects were randomly assigned to different groups using computer-generated random numbers. Halitosis was measured immediately (0 h) after subjects were instructed to use the given test substance in the IET group. In the EDT group, halitosis was measured at 0h, 1h, and 2h after using the given test subtance in the clinical laboratory.

### Study Groups

In the IET group, two subgroups were formed: subjects took a fresh-breath mild effervescent tablet (MT) or a lozenge tablet (LT) 1 tablet, both keeping the tablet in the mouth for 5–10 min. The two tablets had the same active ingredients but different dosage forms. The control group gargled with 10 ml of a fresh-breath mouthwash (MW) for 30 s according to the instructions. Halitosis was measured after complete consumption of the given tablet or gargling (Table 1).

**Table 1:** Groups and sample information

Group	Active ingredients	Instructions for use
Fresh-breath mild effervescent tablet (MT)	ORALIS SB powder*, Champignon concentrate, Licorice extracts	1 tablet, keep it in the mouth for 5–10 min
Fresh-breath lozenge tablet (LT)	ORALIS SB powder *, Champignon concentrate, Licorice extracts	1 tablet, keep it in the mouth for 5–10 min
Mouthwash(MW)	CB12 mouthwash (zinc acetate, sodium fluoride, chlorhexidine diacetate-MEDA OTC AB)	Gargle with 10 ml for 30 s
*ORALIS SB powder is a mixed blend, including Lacticaseibacillus rhamnosus R11, Lactobacillus helveticus R52, Bifidobacterium longum subsp. longum R175, and Saccharomyces boulardii, with an addition of not less than 5.0×10^8^ CFU/tablet during manufacturing.

In the EDT group, there were three subgroups, where 1. participants used commercial fresh-breath probiotic powder (control), 2. participants used commercial fresh-breath probiotic tablets (control), or 3. participants used the fresh-breath mild effervescent tablet (MT; experimental group). The subjects were randomly assigned to these three subgroups. In the first control group, the commercial probiotic powder (PP; Wonder Lab; Shenzhen, China) contained the active ingredients Lactobacillus plantarum, Lactobacillus rhumnosus, Bifidobacterium animalis subsp. lactis, Bifidobacterium longum subsp. longum, Lactobacillus helveticus, and Ligilactobacillus salivarius, with an addition of not less than 4.0×10^10^ CFU/bottle during manufacturing. In the second control group, the commercial probiotic tablets (PT) contained two different strains of Limosilactobacillus reuteri with an addition of not less than 2.0×10^9^ CFU/tablet during manufacturing as active ingredient (BioGaia; Stockholm, Sweden) (Table 3). In the third group (experimental group), EDT of the fresh-breath mild effervescent tablet was carried out, comparing it with the two commercial fresh-breath probiotic products. A total of 30 participants were enrolled, aged 30.21 ±4.06 years. Breath samples were collected at baseline, 0 h, 1 h, and 2 h after consumption of the tablet. Before finishing all the tests, subjects were asked not to eat or contact items with pungent odors or fragrances, but were allowed to drink some water.

**Table 3:** Groups and sample information

Group	Use instruction
Fresh-breath mild effervescent tablet (MT)	1 tablet, keep it in the mouth for 5-10 min
Probiotic powder (PP)	2 bottles, eat directly, swallow water 5-10 s later
Probiotic tablet (PT)	2 tablets, keep them in the mouth for 5-10 min


### Clinical Measurement Tools

#### Questionnaire

The questionnaire included items related to age, marital status, systemic disease, and oral health behaviours and status. Participants answered the questionnaires through a QR code in the recruitment e-mail sent by the research team.

#### Halitosis measurement

One day prior to the measurement, the subjects were instructed not to consume pungent foods to prevent excessive levels of VSC. To prevent cosmetic odors from influencing VSC analysis, the participants were told not to use perfume or cosmetics with fragrance, and female participants were not allowed to wear lipstick on the test day. All subjects were also instructed not to eat, drink, gargle, or brush their teeth for at least 1 h prior to the initial measurements to obtain an initial score that did not vary too widely between subjects. The VSC scores were measured by a portable sulfide monitor OralChroma CHM-2 (FIS; Hyogo, Japan). Volunteers were seated. A sterile disposable syringe was inserted into the oral cavity. The subjects closed their mouths tightly. They were asked to breathe through the nose, while keeping the oral cavity sealed and unventilated for at least 30 s. Subjects had to avoid touching the tip of the syringe with the tongue. After 30 s, the piston was pulled to the very end of the syringe and the syringe was filled with a breath sample from the oral cavity. Then the piston was pushed and the gas returned into the oral cavity. The piston was pulled again to the very end to fill it with another breath sample. The syringe was then removed from the oral cavity. After removing, saliva was wiped from the tip of the syringe with a tissue paper. A volume of 0.5 ml of mouth air in the syringe was injected into the OralChroma CHM-2 in one stroke. Measurements were started automatically and the process was completed after 240 s. The concentrations of the three VSC components (H2S, CH3SH, and CH3SCH3) were displayed in units of either ng/10 ml or ppb (nmol/mol). In this study, concentration of VSC was recorded in ppb.

### Ethics Approval and Consent to Participate

The study was conducted in accordance with the ethical principles of the Declaration of Helsinki and its subsequent amendments. The clinical trial was reviewed by the China Ethics Committee of Registering Clinical Trails, and registered on Chinese Clinical Trial Registry (ChiCTR) (registration date: 17/09/2021; registration number: ChiCTR2100051256, https://www.chictr.org.cn/showproj.html?proj = 129742). An informed written consent containing details of the nature, potential risks and benefits of the study were given to all participants. The study was carried out in the clinical laboratory in a pharmaceutical company (Sirio Pharma; Shantou, China).

### Outcome Assessments

Primary outcome statistical analysis was based on the comparisons of the total amount of VSC (H2S, CH3SH and CH3SCH3) of treatment groups. VSC was measured at baseline, immediately after consuming the test subtance (0 h), 1 h after taking the test substance, and 2 h after taking the test substance. In the secondary analysis, the amounts of three VSC components H2S, CH3SH and CH3SCH3 were compared.

### Statistical Analysis

GraphPad Prism 8.0 Software was used for the statistical analysis. Data were presented as the mean ± standard deviation (SD). Student’s t-test and one-way ANOVA were performed to evaluate H2S, CH3SH, CH3SCH3 and VSCs between pre- and post-intervention stages. In addition, Tukey’s post-hoc test was used to compare differences among different groups at baseline, at treatment timepoint and in the EDT. A p-value <0.05 was used to report the statistical significance of results, and p <0.01 was considered considered highly statistically significant.

## RESULTS

### Immediate Effect on Halitosis 

#### Study population, groups and samples

Thirty-one participants were enrolled, with a mean age of 32.04 ± 3.53 years. No statistically significant differences from baseline status were noticed among the three groups (Table 2).

**Table 2:** Baseline subject characteristics

Characteristics	Mild effervescent tablet (MT), n = 11	Lozenge tablet (LT), n = 10	Mouthwash (MW), n = 10	p-value
Age, years (mean ± SD)	31.70 ± 3.72	32.33 ± 2.79	32.13 ± 3.98	0.93
Gender Male n (%): female n (%)	6 (55%): 5 (45%)	4 (40%):6 (60%)	5 (50%):5 (50%)	0.79
VSCs, ppb (mean ± SD)	482.31 ± 157.30	482.31 ± 157.30	482.31 ± 157.30	0.83
One-way ANOVA and Tukey’s test (p<0.05). Values are means± standard deviations.

#### Immediate effect on VSCs level

**Fig 2:**
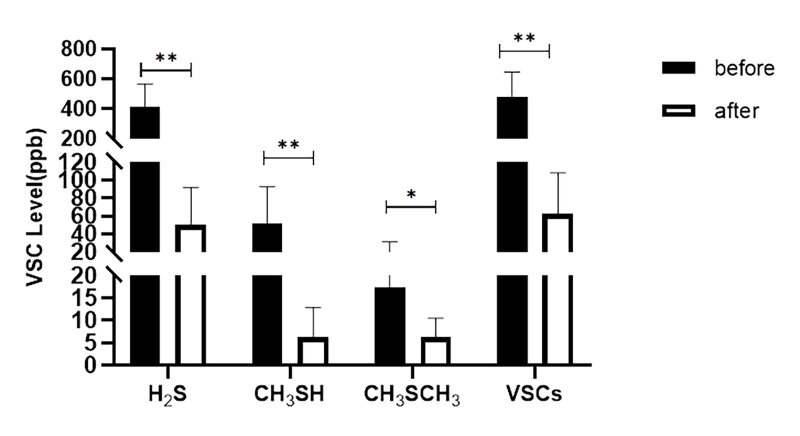
The effect of fresh-breath mild effervescent tablet on VSC level in breath samples.

**Fig 3:**
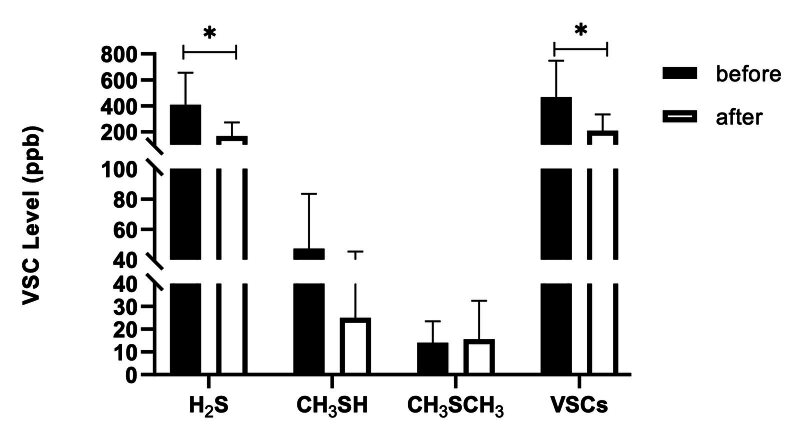
The effect of fresh-breath lozenge on VSC level in breath samples.

**Fig 4:**
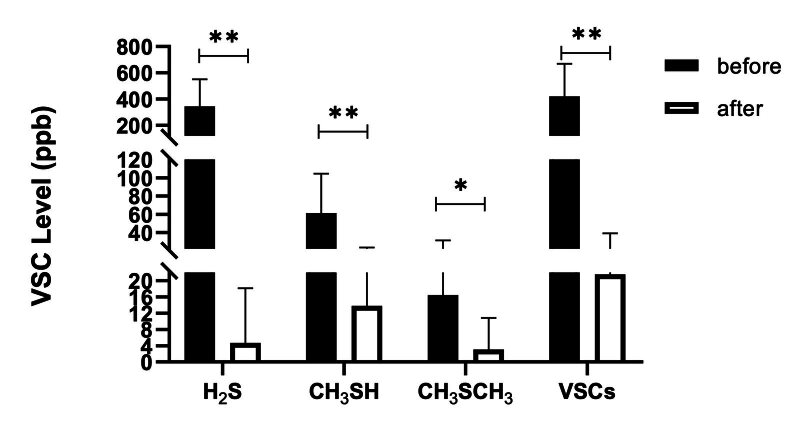
The effect of mouthwash on VSC level in breath samples.

The VCS scores of each group (H2S, CH3SH scores, CH3SCH3) scores and the total VSC scores are illustrated in Figs 2 to 4. Compared to baseline, the fresh-breath mild effervescent tablet can tatistically significantly lowered the level of H2S (p < 0.01), CH3SH (p < 0.01), CH3SCH3 (p < 0.05) and VSCs (p < 0.01). The fresh-breath lozenge statistically significantly lowered the level of H2S (p < 0.05) and VSCs (p < 0.05). The levels of H2S (p < 0.01), CH3SH (p < 0.01), CH3SCH3 (p < 0.05) and VSCs (p < 0.01) were statistically significantly lower in the mouthwash group.

**Fig 5:**
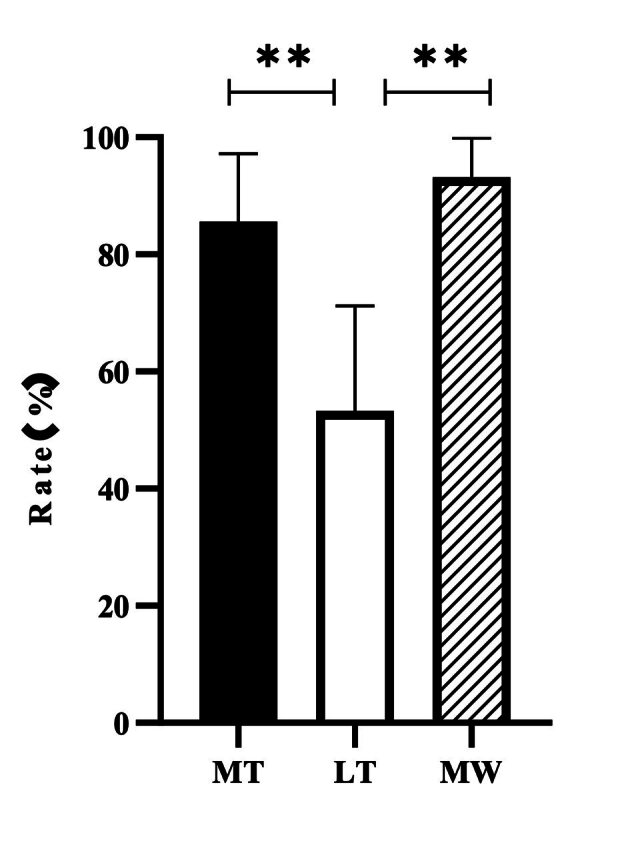
The effect of different groups on VSC level.

The comparison of the total VSCs scores of these three groups were shown in Fig 5. Both the MT (p < 0.01) and MW (p < 0.01) groups had a better effect on VSC reduction compared to the LT group (Fig 4). However, there was no statistically significant difference in anti-halitosis between MT and MW groups (p = 0.38). These groups had the same active ingredients but with different dosage forms, but the efficacy of MT and LT was totally different. The mild effervescent tablet was more effective than the lozenge in terms of reducing VSC. The fresh-breath mild effervescent tablet had a similar effect vs the CB12 mouthwash on anti-halitosis.

### Duration Effect on Halitosis 

The baseline characteristics of the subjects in the three EDT groups showed no statistically significant difference (Table 4).

**Table 4:** Baseline subject characteristics

Characteristics	Mild effervescent tablet (MT), n = 10	Probiotic powder (PP), n = 10	Probiotic tablet (PT), n = 10	p-value
Age, years (mean ± SD)	29.80 ± 3.57	29.44 ± 4.11	31.30 ± 4.24	0.59
Female, n (%)	6(60%):4(40%)	5(50%):5(50%	6(60%):4(40%)	0.87
VSCs, ppb (mean ± SD)	507.82 ± 314.69	492.10 ± 430.24	418.99 ± 196.00	0.77
One-way ANOVA and Tukey’s test(p<0.05). Values are means± standard deviations.

#### Duration Effects on VSC Level

**Fig 6:**
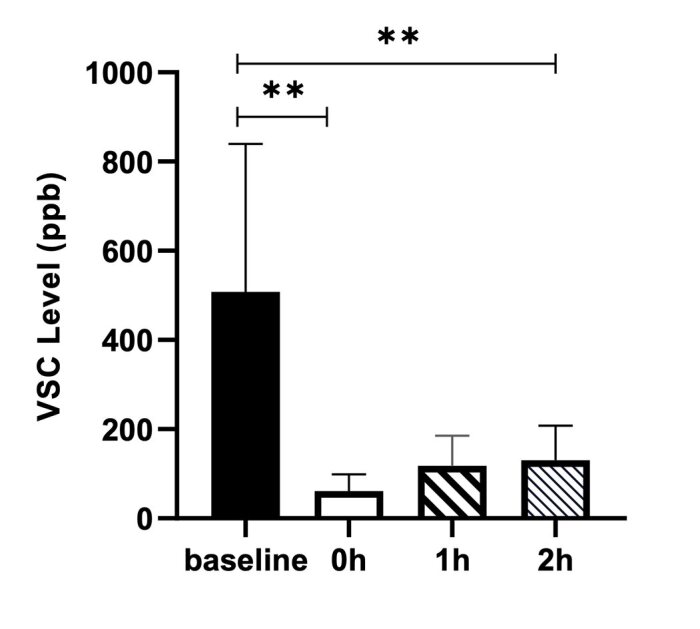
The effective duration for fresh-breath mild effervescent tablet.

The results of the VSC scores of the test group in each measurement period are presented in Fig 6. The fresh-breath mild effervescent tablet had an immediate effect on VSCs level (p < 0.01), which was consistent with the IET. The VSCs level was still significantly lower than the baseline 1 h (p < 0.01) and 2 h (p < 0.01) later after consumption (Fig 6).

**Fig 7:**
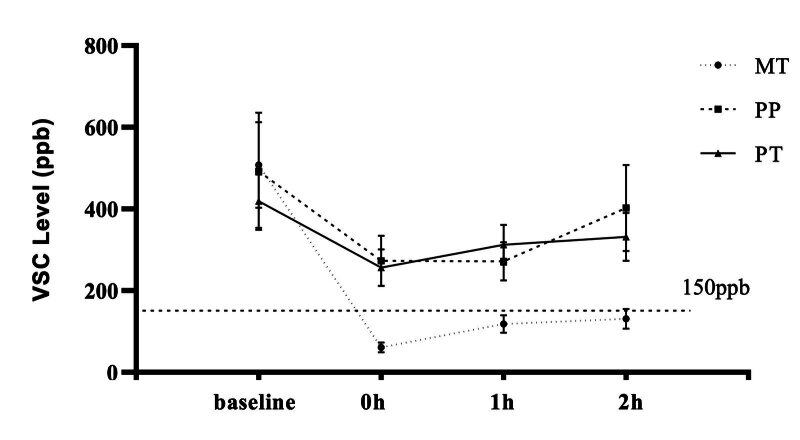
Comparison of effective duration for fresh breath of different fresh-breath products.

The comparison of the VSC scores of the test group and two control groups in each measurement period is presented in Fig 7. The two control groups did not show any statistically significant difference compared to baseline, only a slight decrease at 0 h (PP group, p = 0.19; PT group, p = 0.06). The VSCs level of these two groups gradually reverted to the baseline level, whereas the VSC of the mild effervescent tablet group was still lower than 150 ppb (Fig 7).

## DISCUSSION

There are many ways to combat halitosis, such as daily toothbrushing and the use of mouthwash. Mouthwashes may have a good but temporary effect on bad breath. However, most of mouthwashes contain antibacterial agents, such as chlorhexidine, which may kill both the harmful and the good bacteria in the mouth. Therefore, long-term use of mouthwashes could have detrimental effects on the healthy microbiome.^3^

In this study, the fresh-breath mild effervescent tablet was explored as an alternative to mouthwash for treatment of halitosis. Natural extracts (champignon concentration, licorice extracts) in the tablet have an immediate anti-halitosis effect. The champignon component in the sample is a mixed blend of the mushroom Agaricus bisporus and green coffee bean extract. The mushroom Agaricus bisporus can convert the mercapto group of methyl mercaptan to a sulfo group, thereby inhibiting the odour.^21^ Green coffee bean extract contains chlorogenic acid, which Yadav et al^24^ and Bharath et al^4^ showed can statistically significantly reduce the salivary Streptococcus mutans counts, and inhibit periodontopathogenic bacteria Porphyromonas gingivalis, Prevotella intermedia, Fusobacterium nucleatum, and Aggregatibacter actinomycetemcomitans. Glycyrrhizic acid and glycyrrhizin flavonoids in licorice have a good inhibitory effect on a variety of periodontal pathogens, and can help prevent dental diseases such as caries, periodontitis, gingivitis, candidiasis and periodontitis.^6^ ORALIS SB powder is a mixed probiotic blend containing Lacticaseibacillus rhamnosus R11, Lactobacillus helveticus R52, Bifidobacterium longum subsp. longum R175, and Saccharomyces boulardii, active ingredients which have been clinically confirmed to reduce salivary Streptococcus mutans counts, inhibit dental plaque, reduce gingivitis and promote a healthy microbiome.^11,12,15,23^ Probiotics, an active ingredient for oral microbial colonisation, would seem to be effective against halitosis in the long-term. Streptococcus salivarius subsp. thermophilus K12 can relieve bad breath but needs to be applied via mouthwash once a day for 2 weeks.^10^ In the EDT of this study, the two control probiotic groups did not show an immediate effect on VSC level. Although the VSC level of the two control probiotic groups decreased at 0 h compared with the baseline, the authors deemed that it was a temporary effect of saliva-flow cleaning during consumption of the test substance, and showed no statistically difference. The VSC level gradually recovered to the initial value of the two control groups, while in the mild effervescent tablet group, VSC level was still statistically significantly lower 2 h after consumption (p < 0.01).

It was interesting to find that despite the same active ingredients, the dosage form “effervescent tablet” had a better effect on VSC level than the lozenge dosage form. Salivation is stimulated through the acid-base agent in the mild effervescent tablet during oral administration. Saliva flow plays an important role in the formation of oral malodor. For instance, Kleinberg et al^17^ found that saliva is a major source of oxygen for the oral bacteria. Rapid saliva flow provides greater availability of oxygen and less opportunity for salivary peptides and proteins to be degraded by the oral bacteria.^18^ Furthermore, saliva flow can continuously provide self-cleaning and wash away pathogenic bacteria which prevents malodour production. Therefore, mild effervescent tablets were more effective than the lozenges in terms of freshening the breath.

With the present and somewhat encouraging findings on halitosis, it could be of interest to further explore probiotic-based products. The primary treatment for oral malodour is the reduction of bacterial populations, especially those present on the tongue, by use of a variety of antimicrobial agents or mechanical devices. However, shortly after treatment, the problematic bacteria quickly repopulate the tongue and the malodour returns. Probiotics could therefore offer a complementary and more long-term treatment strategy to combat bad breath.^5^ Whether or not the bacterial composition was altered in the present study remains an open question, but long-term studies are needed to evaluate the microbial profile of oral microbiota, with special emphasis on non-VSC producing bacteria, to further elucidate the research question.

## CONCLUSION

The fresh-breath mild effervescent tablet had an immediate effect on VSC level which could last for more than 2 h, and was considered to be a healthier alternative than mouthwash.

The natural extracts in the mild effervescent tablet might play a temporary role in freshening the breath by lowering the VSC level. However, inhibiting pathogenic bacteria growth and promoting heathy oral status through a good oral microecological balance may be a longer-term and more effective way to prevent oral malodor. Further study of the effects of the fresh-breath mild effervescent tablet on the oral microecological environment in subjects with halitosis could provide more comprehensive results.

Probiotic-based products may have a potential application as an adjunct to oral hygiene improvement and antibacterial rinses for the management of oral malodour. Given the mis- and over-use of antimicrobials, probiotic plus therapy could be a promising alternative to combat infections by using harmless bacteria to displace pathogenic microorganisms.
